# Hydrogen Sulfide Donor Protects Porcine Oocytes against Aging and Improves the Developmental Potential of Aged Porcine Oocytes

**DOI:** 10.1371/journal.pone.0116964

**Published:** 2015-01-23

**Authors:** Tereza Krejcova, Miroslava Smelcova, Jaroslav Petr, Jean-Francois Bodart, Marketa Sedmikova, Jan Nevoral, Marketa Dvorakova, Alena Vyskocilova, Ivona Weingartova, Veronika Kucerova-Chrpova, Eva Chmelikova, Lenka Tumova, Frantisek Jilek

**Affiliations:** 1 Czech University of Life Sciences in Prague, Faculty of Agrobiology, Food and Natural Resources, Department of Veterinary Sciences, Prague, Czech Republic; 2 Institute of Animal Science, Prague, Czech Republic; 3 Université Lille1, Sciences et Technologies, Laboratoire de Régulation des Signaux de Division – EA 4479, Villeneuve d´Ascq, France; Institute of Zoology, Chinese Academy of Sciences, CHINA

## Abstract

Porcine oocytes that have matured in in vitro conditions undergo the process of aging during prolonged cultivation, which is manifested by spontaneous parthenogenetic activation, lysis or fragmentation of aged oocytes. This study focused on the role of hydrogen sulfide (H_2_S) in the process of porcine oocyte aging. H_2_S is a gaseous signaling molecule and is produced endogenously by the enzymes cystathionine-β-synthase (CBS), cystathionine-γ-lyase (CSE) and 3-mercaptopyruvate sulfurtransferase (MPST). We demonstrated that H_2_S-producing enzymes are active in porcine oocytes and that a statistically significant decline in endogenous H_2_S production occurs during the first day of aging. Inhibition of these enzymes accelerates signs of aging in oocytes and significantly increases the ratio of fragmented oocytes. The presence of exogenous H_2_S from a donor (Na_2_S.9H_2_O) significantly suppressed the manifestations of aging, reversed the effects of inhibitors and resulted in the complete suppression of oocyte fragmentation. Cultivation of aging oocytes in the presence of H_2_S donor positively affected their subsequent embryonic development following parthenogenetic activation. Although no unambiguous effects of exogenous H_2_S on MPF and MAPK activities were detected and the intracellular mechanism underlying H_2_S activity remains unclear, our study clearly demonstrates the role of H_2_S in the regulation of porcine oocyte aging.

## Introduction

Porcine oocytes, similarly to the majority of mammal oocytes, can be fertilized in the MII stage of meiotic maturation. If oocytes are not fertilized shortly after the completion of meiotic maturation, then they undergo a number complex undesirable changes called aging [1,2]. Their quality and capacity to undergo proper further embryonic development after fertilization rapidly decrease [3].

Oocytes undergo functional and morphological changes during aging. Among other contributing factors, oocyte aging is partly due to changes in M-phase promoting factor (MPF) and mitogen-activated protein kinase (MAPK) activity, which are necessary to maintain meiotic arrest in metaphase II [4,5]. Diminution of MAPK activity and MPF inactivation leads to one of the main manifestations of aging: spontaneous parthenogenetic activation. Aged oocytes may also undergo fragmentation (apoptosis) induced by a high level of MAPK activity, or lysis [6–9].

Hydrogen sulfide (H_2_S) is one of the upstream factors that control MAPK activity [10]. H_2_S, a gaseous mediator, is produced in cells from the amino acid L-cysteine by three enzymes: cystathionine-β-synthase (CBS), cystathionine-γ-lyase (CSE) and 3-mercaptopyruvate sulfurtransferase (MPST). The expression and activity of these enzymes vary in different tissues [11,12]. The expression of these enzymes and endogenous H_2_S production from tens to hundreds of micromoles have been described in the central nervous and the respiratory system [13–15]. H_2_S is also involved in the regulation of reproduction.

CBS and CSE expression, but not MPST, have been reported in mouse, rat and human reproductive systems [16,17]. CBS knockout mice have reduced quantities of growing follicles and irregular, shorter estrus cycles [18,19]. Liang et al. [20] demonstrated the presence of CBS in follicular and granulose cells but not in oocyte alone. However, decreased CBS expression in granulose cells has been associated with the inhibition of meiotic maturation in mouse oocytes [21].

The requirement for H_2_S production by cumulus cells for proper porcine oocyte meiotic maturation has been described by Nevoral et al. [22]. H_2_S, by regulating ion channels and kinase activities, participates in the regulation of apoptosis in somatic cells. Its effect can be pro-apoptotic or anti-apoptotic depending on the situation and type of cell [23–26].

We hypothesized that endogenous production of H_2_S is involved in the regulation of porcine oocyte aging and that oocyte aging can be affected by exogenous H_2_S. The aim of this study was to detect the endogenous production of H_2_S in porcine oocytes and to assess its involvement in oocyte aging. Additional aims of the study were to determine whether H_2_S participates in the regulation of MPF and MAPK activities, including whether exogenous H_2_S can suppress the manifestations of aging and improve the quality of aged oocytes in relation to consecutive embryonic development.

## Materials and Methods

### Collection and Cultivation of Oocytes

Porcine ovaries were obtained from a local slaughterhouses in Cesky Brod and Pilsen from gilts (Large White × Landrace, slaughter weight 110 kg, 6 months old) during an unknown stage of the oestrous cycle and were transported to the laboratory in a saline solution (0.9% natrium chloride) at 39°C. Oocytes were obtained through the aspiration of follicles (2 to 5 mm in diameter) with a 20-gauge needle. Only oocytes with compact cumuli were chosen for experiments. Oocytes were washed three times in the culture medium before cultivation. Oocytes were cultivated in a modified M199 medium (GibcoBRL, Life Technologies, Carlsbad, USA) containing sodium bicarbonate (32.5 mM; Sigma-Aldrich, USA), calcium L-lactate (2.75 mM; Sigma-Aldrich, USA), sodium pyruvate (0.25 mg/ml; Sigma-Aldrich, USA), gentamicin (0.025 mg/ml; Sigma-Aldrich, USA), HEPES (6.3 mM; Sigma-Aldrich, USA), 10% (v/v) foetal calf serum (Gibco BRL, Life Technologies, Germany) and 13.5 IU eCG: 6.6 IU hCG/ml (P.G. 600, Intervet, Boxmeer, Netherlands).

The oocytes were then cultured in 3.5 cm diameter Petri dishes (Nunc, Roskilde, Denmark) containing 3 ml of culture medium at 39°C. The Petri dishes were placed into incubator in a mixture of 5% CO_2_ in air for 48 hours, until oocytes reached the metaphase of the second meiotic division (MII). Subsequently, oocytes were denuded of cumular cells by repeated pippeting through a thin glass capillary and then cultivated according to specific experimental procedures.

### *In Vitro* Cultivation of Aging Oocytes with H_2_S Donor and Inhibitors of H_2_S-producing Enzymes

After denuding the oocytes in metaphase II, they were exposed to prolonged cultivation (aging) in a modified medium M199 (GibcoBRL, Life Technologies, Carlsbad, USA) without P.G. 600. Once again oocytes were cultivated in 3.5 cm diameter Petri dishes (Nunc, Roskilde, Denmark), containing 3 ml of culture medium at 39°C. The Petri dishes were placed into an incubator in a mixture of 5% CO_2_ in air for 24, 48 and 72 hours. The hydrogen sulfide donor, Na_2_S.9H_2_O (Sigma-Aldrich, USA), in its effective concentrations was used. Effective concentrations of this H_2_S donor were selected after the previous testing. To monitor the effect of elevated levels of H_2_S on porcine oocytes aging, the concentration 300 μM of Na_2_S.9H_2_O was selected because the fragmentation of the porcine oocytes cultivated in medium supplemented with such concentration of Na_2_S.9H_2_O after 72 hours failed to occur as well as the highest number of oocytes were still in metaphase II (see Supporting Information, S1 Table).

Specific inhibitors were used to inhibit the enzymes activity. We used 1 mM oxamic acid (OA; Sigma-Aldrich, USA) as a CBS inhibitor, 1 mM beta-kyano-L-alanine (KA; Sigma-Aldrich, USA) as a CSE inhibitor and 5mM alpha-ketoglutaric acid disodium salt dihydrate (KGA; Sigma-Aldrich, USA) as a MPST inhibitor. These inhibitors were used alone or in various combinations (1 mM OA + 1 mM KA; 1 mM OA + 1 mM KGA; 1 mM KA + 5 mM KGA; 1 mM OA + 1 mM KA + 5 mM KGA). Each concentration of the inhibitors was selected after the previous testing (data not shown). Experiments were repeated three times and a minimum of 120 oocytes were evaluated in each experiment.

### Parthenogenetic Activation of Oocytes

Oocytes were activated using calcium ionophore A23187 (25 μM, 5 min; Sigma-Aldrich, USA) combined with a 6-dimethyl aminopurine - 6-DMAP (2 mM, 2 h; Sigma-Aldrich, USA) treatment [27]. In experimental groups, porcine oocytes were exposed to prolonged cultivation (aging) for 24 hours in medium M199 supplemented with hydrogen sulfide donor, Na_2_S.9H_2_O in concentrations 150 μM, or 300 μM. These concentrations were selected after previous testing, because in the group of porcine oocytes aged 72 hours in medium supplemented with 150 μM, or 300 μM of Na_2_S.9H_2_O, fragmented oocytes failed to occur (see Supporting Information, S1 Table).

Parthenogenetically activated oocytes were subsequently cultivated 24 hours. Oocytes with formed pronuclei were considered parthenogenetically activated. Experiments were repeated three times and a minimum of 120 oocytes were evaluated in each experiment.

### *In Vitro* Cultivation of Parthenogenetic Embryos

Parthenogenetically activated oocytes were cultured in a NCSU 23 medium [28] in 4-cell Petri dishes (Nunc, Roskilde, Denmark) containing 1 ml of culture medium at 39°C. The Petri dishes were placed into incubator in a mixture of 5% CO_2_ in air. The ratio of embryo cleavage was determined after 48 hours of culture. The ratio of embryos in the morula and blastocyst stage was determined after 168 hours (7 days) of culture. Experiments were repeated three times and a minimum of 120 oocytes were evaluated in each experiment.

### Evaluation of Oocytes and Parthenogenetic Embryos

**Figure 1:**
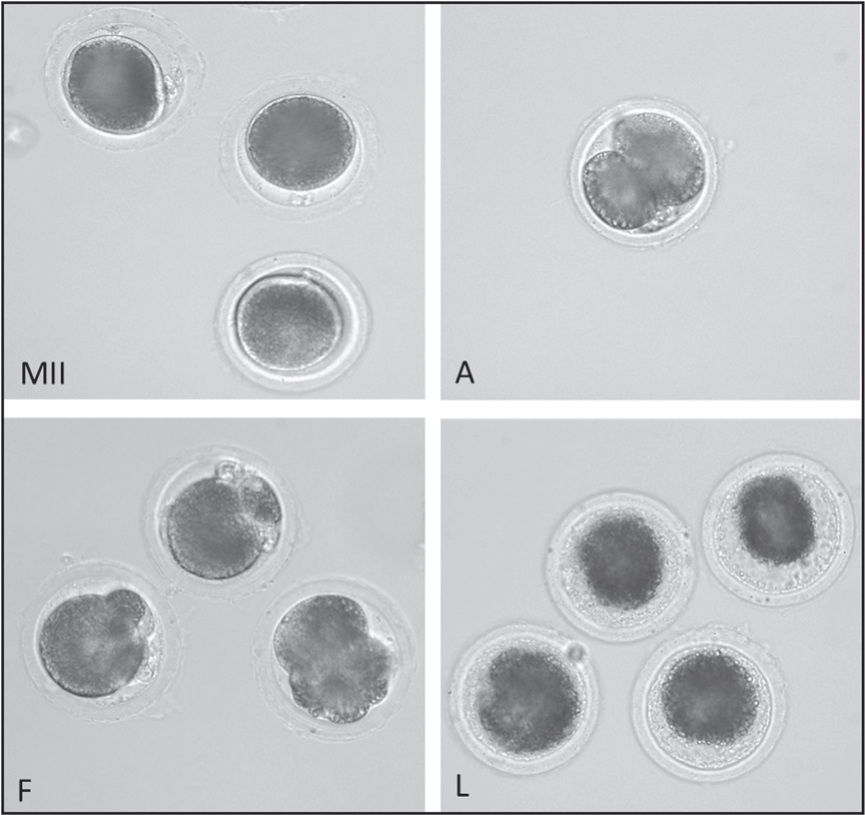
Effect of aging on morphology of porcine oocyte. MII—intact oocyte at metaphase II, A—activated oocyte, F—fragmented oocyte, L—lysed oocyte.

At the completion of the cultivation period, the oocytes and embryos were fixed in an ethanol and acetic acid (3:1, v/v) for 48 hours. Subsequently, fixed oocytes and embryos were stained with 1.0% (w/v) orcein in 50% aqueous-acetic acid. Oocytes and embryos were then examined under a phase-contrast microscope. Aged oocytes were classified into four groups: intact oocytes (oocytes at metaphase II, anaphase II or telophase II); activated oocytes (oocytes with pronuclei or embryos); fragmented oocytes (oocytes were designated as fragmented when fragmented “vesicles” were observed under the *zona pellucida*), and lysed oocytes (rupture of the cytoplasmic membrane and loss of the integrity of the oocyte were the criteria for lysis) (see Fig. 1; [1]).

### Measurement of Hydrogen Sulfide Production

H_2_S production in porcine oocytes was verified through spectrophotometric analysis of S^2−^ ions in accordance with Stipanuk and Beck [29], modified by Abe and Kimura [30] and Pan et al., [31], modified for porcine oocytes. Samples from oocytes at metaphase II, as well as samples from oocytes exposed to aging for 24 hours were prepared for this experiment. Each sample comprised 100 oocytes.

Initially, oocytes were mechanically disrupted in the presence of the following reaction mixture (500 μl) under gaseous nitrogen N_2_ and low temperature (4°C) conditions: pyridoxal 5-phosphate (0.2 M, 5 μl, Sigma-Aldrich, USA) L-cystein (10 mM, 50 μl, Sigma-Aldrich, USA) and deionized water (445 μl).

Subsequently, zinc acetate (1%, 250 μl, Sigma-Aldrich, USA) was added into the reaction mixture. The reaction of the enzymes was initiated by increasing the temperature of the tube from 4°C to 37°C. After the culture (60 min), 50% trichloroacetic acid (250 μl, Sigma-Aldrich, USA) was added to the reaction mixture to stop the reaction. Subsequently, the reaction mixture was incubated for the next 60 minutes at 37°C. Afterwards, the N,N dimethyl-p-phenylenediamine sulphate (20 mM in 7.2 M HCl, 133 μL, Sigma-Aldrich, USA) and FeCl_3_ (30 mM in 1.2 M HCl, 133 μL, Sigma-Aldrich, USA) were added. The sample absorbance was measured by a spectrophotometer at 670 nm. The results of these measurements were presented as a ratio relative to the group of oocytes at metaphase II, which was taken as a value of 100%.

### Histone H1 and Myelin Basic Protein Double Assay

Histon H1 kinase assay and MBP (Myelin basic protein) were carried out to determine MPF and MAPK activity by measuring their capacity to phosphorylate their substrates (histon H1 and MBP—Myelin basic protein) [22]. Histon H1 kinase and MBP assays were determined in oocytes at metaphase II, oocytes aged for 12 and 24 hours in modified M199 medium, oocytes aged for 12 and 24 hours in modified M199 medium supplemented with 300 μM of Na_2_S.9H_2_O and oocytes aged for 12 and 24 hours in modified M199 medium supplemented with 1 mM Oxamic acid + 1 mM beta-kyano-L-alanine + 5 mM alpha-ketoglutaric acid disodium salt dihydrate. Samples were immediately frozen to −80°C. The data obtained were analysed and evaluated by MultiGauge 2.0 software. The results were presented as the ratio relative to the activity of group of oocytes at metaphase II, which was taken as a value 1.

### Statistical analysis

Data from all the experiments was subjected to statistical analysis. The SAS 9.0 software (SAS Institute Inc., USA) was used for statistical analysis and data. Significant differences between groups were determined using the ANOVA test. An a P value of less than 0.05 was considered significant.

### Ethics Statements

All animal work was conducted according to Act No 246/1992 Coll., on the protection of animals against cruelty under supervision of Central Commission for Animal Welfare, approval ID 018/2010. The approval of Institutional Animal Care and Use Commitee (IACUC) was not required as the experimens were performed under in vitro conditions.

## Results

### Endogenous Production of H_2_S Decreases During the First 24 hours of Aging

**Figure 2:**
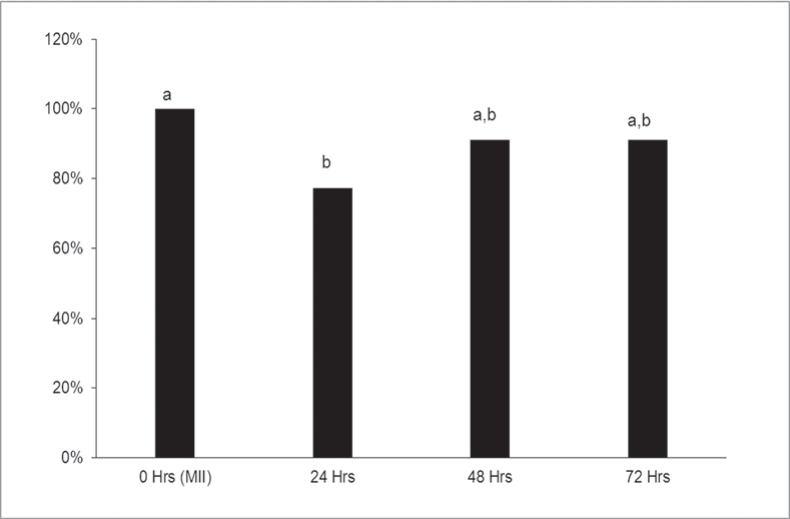
Determination of endogenous H_2_S production during porcine oocyte aging. Oocytes were cultivated to metaphase II (MII). Hydrogen sulfide production was carried out using the spectrophotometric method. MII oocytes, as well as oocytes exposed to prolonged cultivation for 24, 48 and 72 hours, were examined. The results of the measurement are presented as a ratio relative to the MII oocyte group. ^*a, b*^ *Statistically significant differences in spontaneous hydrogen sulfide production are indicated by different superscripts (P<0.05). Each experiment was repeated four times. The total number of oocytes in each sample was 100*.

In this experiment, we focused on evaluating endogenous H_2_S production during the porcine oocyte aging process. We demonstrated that H_2_S is enzymatically produced in porcine oocytes through H_2_S producing enzyme activity. The highest level of H_2_S production occurred in mature oocytes that were not subjected to aging. Endogenous production of H_2_S significantly decreased by 29% as early as at 24 hours of oocyte aging compared with mature oocytes in metaphase II that were not subjected to aging (see Fig. 2).

### Exogenous H_2_S Protects Oocytes Against Aging

In the next experiment, we attempted to influence the signs of aging by compensating for the decrease in H_2_S production with exogenous H_2_S by cultivating the oocytes in a medium containing an H_2_S donor.

A gradual, significant increase in the proportion of spontaneously parthenogenetically activated and fragmented oocytes was monitored during porcine oocyte aging *in vitro*. Oocytes with pronuclei or embryos were classified as parthenogenetically activated and oocytes with fragmented “vesicles” under the *zona pellucida* were classified as fragmented. Oocyte at MII, AII and TII were considered intact (see Fig. 1). After 48 hours of aging, 37.2 ± 2.5% of the oocytes were parthenogenetically activated and 18.3 ± 2.9% were fragmented in the control group; after 72 hours of aging, 47.5 ± 2.5% of the oocytes were parthenogenetically activated and 25.9 ± 2.9% were fragmented.

**Figure 3:**
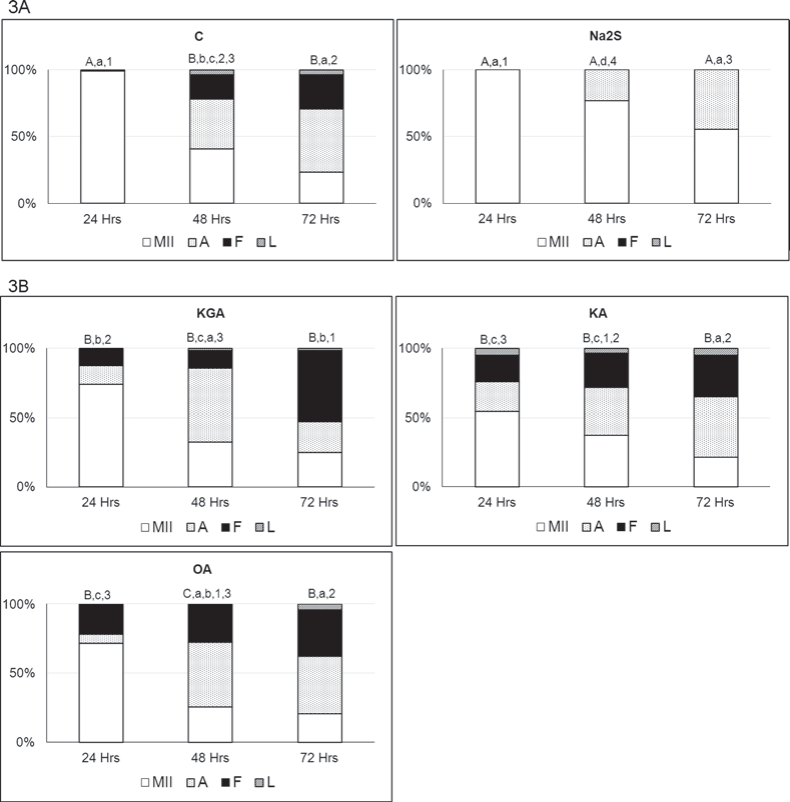
Effects of an elevated H_2_S level and inhibition of H_2_S producing enzymes during oocyte aging. Oocytes were cultivated to metaphase II and then exposed to prolonged cultivation in a modified M199 medium for 24, 48 and 72 hours in the presence of a H_2_S donor or H_2_S producing enzymes inhibitors. **3A**. Na_2_S (Na_2_S.9H_2_O; 300 μM) was used as the H_2_S donor. **3B**. Oxamic acid (1mM, OA) was used as a CBS inhibitor, beta-kyano-L-alanine (1mM, KA) was used as a CSE inhibitor and alpha-ketoglutaric acid disodium salt dihydrate (5mM, KGA) was used as a MPST inhibitor. *C- control; Na_2_S (300 μM, Na_2_S.9H_2_O); KGA—alpha-ketoglutaric acid disodium salt dihydrate (5 mM); KA – beta-kyano-L-alanine (1mM); OA—oxamic acid (1 mM); MII—intact oocytes (oocytes at metaphase II, anaphase II or telophase II), A—activated oocytes (oocytes with pronuclei or embryos), F—fragmented oocytes, L—lysed oocytes. Different letters and numbers indicate significant differences between different treatments and hours of aging (P<0.05). A,B,C – statistically significant differences in portion of MII stage oocytes between individual treatments. a,b,c,d – statistically significant differences in portion of activated oocytes between individual treatments. 1,2,3 – statistically significant differences in portion of fragmented oocytes between individual treatments*.

The presence of H_2_S donor (300 μM Na_2_S.9H_2_O) in the culture medium completely suppressed the fragmentation of aging oocytes. After 48 hours of aging, only intact oocytes (76.7 ± 3.8%) and parthenogenetically activated oocytes (23.3 ± 3.8%) were observed in the experimental group. Fragmented oocytes were not detected in the experimental group, even after 72 hours of aging (55.0 ± 5.0% intact oocytes and 45.0 ± 5.0% parthenogenetically activated oocytes) (Fig. 3A, for detailed results see Supporting Information S2, S3, S4 Tables).

### Inhibition of Endogenous H_2_S Production Accelerates the Oocyte Aging Process

In this experiment, we focused on monitoring the effects of inhibitors of individual enzymes (CBS, CSE, MPST) that are responsible for the endogenous production of H_2_S. Inhibition of these enzymes led to earlier onset of signs of aging in porcine oocytes.

Oocytes that were aged in medium containing inhibitors, in contrast to the control group, were already parthenogenetically activated or fragmented during the first 24 hours of aging. In contrast, 100% of the oocytes in the control group were in metaphase II during the first 24 hours of aging (Fig. 3A), and among oocytes cultured in the presence of alpha-ketoglutaric acid disodium salt dihydrate (inhibitor of MPST), 13.3 ± 2.9% of the oocytes were parthenogenetically activated and 12.5 ± 0.0% were fragmented. In the group of oocytes cultured in the presence of beta-kyano-L-alanine (inhibitor of CSE) and oxamic acid (inhibitor of CBS), 7.5 ± 2.5% and 6.6 ± 1.4% of the oocytes were parthenogenetically activated and 20.0 ± 2.5% and 21.7 ± 3.8% were fragmented, respectively.

The effect of inhibitors decreased gradually after 48 and 72 hours of oocyte aging. After 72 hours of aging, we observed a significant difference in comparison to the control group only in the group of oocytes treated with MPST inhibitor (47.5 ± 2.5% vs 22.0 ± 3.5% parthenogenetically activated oocytes and 25.9 ± 2.9% vs 51.3 ± 4.3% fragmented oocytes) (Fig. 3B). For detailed results, refer to Supporting Information S2, S3, S4 Tables.

### Inhibition of Endogenous H_2_S Production Can Be Reversed by Exogenous H_2_S

The specific effect of inhibitors was verified by the reversion of this inhibitory effect using the H_2_S donor Na_2_S.9H_2_O at a concentration of 300 μM.

**Figure 4:**
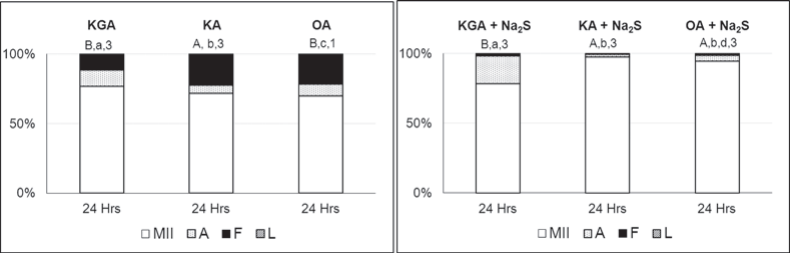
Reversion of the effects of CBS, CSE and MPST inhibitors using a H_2_S donor. Oocytes were cultivated to metaphase II and then exposed to prolonged cultivation (24 hours) in a modified M199 medium supplemented with a H_2_S donor (Na_2_S.9H_2_O; 300 μM) and the following individual inhibitors: oxamic acid (1mM, OA), beta-kyano-L-alanine (1mM, KA), and alpha-ketoglutaric acid disodium salt dihydrate (5mM, KGA). *Na_2_S (300 μM, Na_2_S.9H_2_O); KGA—alpha-ketoglutaric acid disodium salt dihydrate (5 mM); KA – beta-kyano-L-alanine (1mM); OA—oxamic acid (1 mM); MII—intact oocytes (oocytes at metaphase II, anaphase II or telophase II), A—activated oocytes (oocytes with pronuclei or embryos), F—fragmented oocytes, L—lysed oocytes; Different letters and numbers indicate significant differences between different treatments and hours of aging (P<0.05). A,B – statistically significant differences in portion of MII stage oocytes between individual treatments. a,b,c,d – statistically significant differences in portion of activated oocytes between individual treatments. 1,2,3 – statistically significant differences in portion of fragmented oocytes between individual treatments*.

The effect of CBS and CSE inhibitors could be completely reversed by the addition of H_2_S donor. The H_2_S donor reversed the effect of the MPST inhibitor but only in the presence of fragmented oocytes (Fig. 4). For detailed results, refer to Supporting information S5 Table.

### Effect of Double or Triple Inhibition of H_2_S-Producing Enzymes Can Be Reversed by Exogenous H_2_S

Subsequently, we focused on the possibility of supplementing endogenous H_2_S production with an exogenous donor to determine the effect on oocyte aging. We studied the effect of H_2_S donor on oocyte aging after inhibiting two or all three H_2_S-producing enzymes.

Although the effects of a combination of inhibitors differed from one another, in all of the experimental groups, the presence of exogenous H_2_S significantly suppressed the fragmentation of aging oocytes. Fragmentation was completely suppressed by the application of exogenous H_2_S in the group of oocytes that were aged in the presence of two inhibitors: inhibitors of CBS and CSE (0.0% vs 25.8 ± 3.8%; 72 hours of aging) and inhibitors of CSE and MPST (0.0% vs 49.2 ± 1.4%; 72 hours of aging). The effect of inhibition by CBS and MPST together was partially reversed by the application of exogenous H_2_S (10.0 ± 0.0% vs 24.2 ± 3.8%; 72 hours of aging).

**Figure 5:**
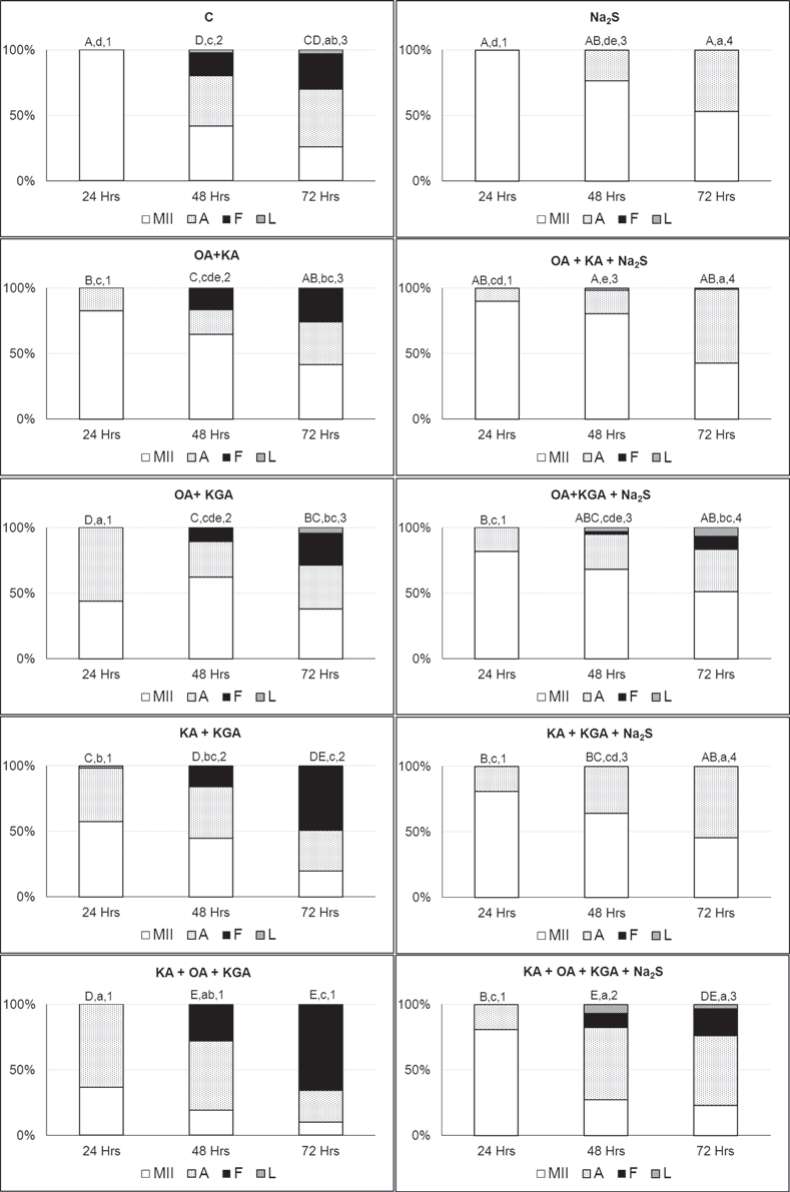
Effects of concurrent CBS, CSE and MPST inhibition and its reversion using a H_2_S donor. Oocytes were cultivated to metaphase II and then exposed to prolonged cultivation in a modified M199 medium supplemented with a H_2_S donor (Na_2_S.9H_2_O; 300 μM) and the inhibitors for 24, 48 and 72 hours. Various combinations of oxamic acid (1mM, OA) which was used as a CBS inhibitor, beta-kyano-L-alanine (1mM, KA) which was used as a CSE inhibitor and alpha-ketoglutaric acid disodium salt dihydrate (5mM, KGA) which was used as a MPST inhibitor were used in this experiment. To reverse effects of inhibitors, a H_2_S donor (300 μM, Na_2_S.9H_2_O) was added to each experimental group. *C- control; Na_2_S (300 μM, Na_2_S.9H_2_O); KGA—alpha-ketoglutaric acid disodium salt dihydrate (5 mM); OA—oxamic acid (1 mM); KA – beta-kyano-L-alanine (1mM); MII—intact oocytes (oocytes at metaphase II, anaphase II or telophase II), A—activated oocytes (oocytes with pronuclei or embryos), F—fragmented oocytes, L—lysed oocytes; Different letters and numbers indicate significant differences between different treatments and hours of aging (P<0.05). A,B,C,D – statistically significant differences in portion of MII stage oocytes between individual treatments. a,b,c,d,e – statistically significant differences in portion of activated oocytes between individual treatments. 1,2,3,4 – statistically significant differences in portion of fragmented oocytes between individual treatments*.

Simultaneous inhibition of all three H_2_S-producing enzymes led to a significant detrimental effect on oocyte aging with the largest proportion of parthenogenetically activated and fragmented oocytes. Although the reversal of this inhibitory effect by the H_2_S donor had a reduced efficiency in this case, it was significant (20.5 ± 4.0% vs 65.8 ± 2.9%; 72 hours of aging) (see Fig. 5; Supporting Information S6, S7, S8 Tables).

### MPF and MAPK Activity is Influenced by the H_2_S Level in Aging Oocytes

**Figure 6:**
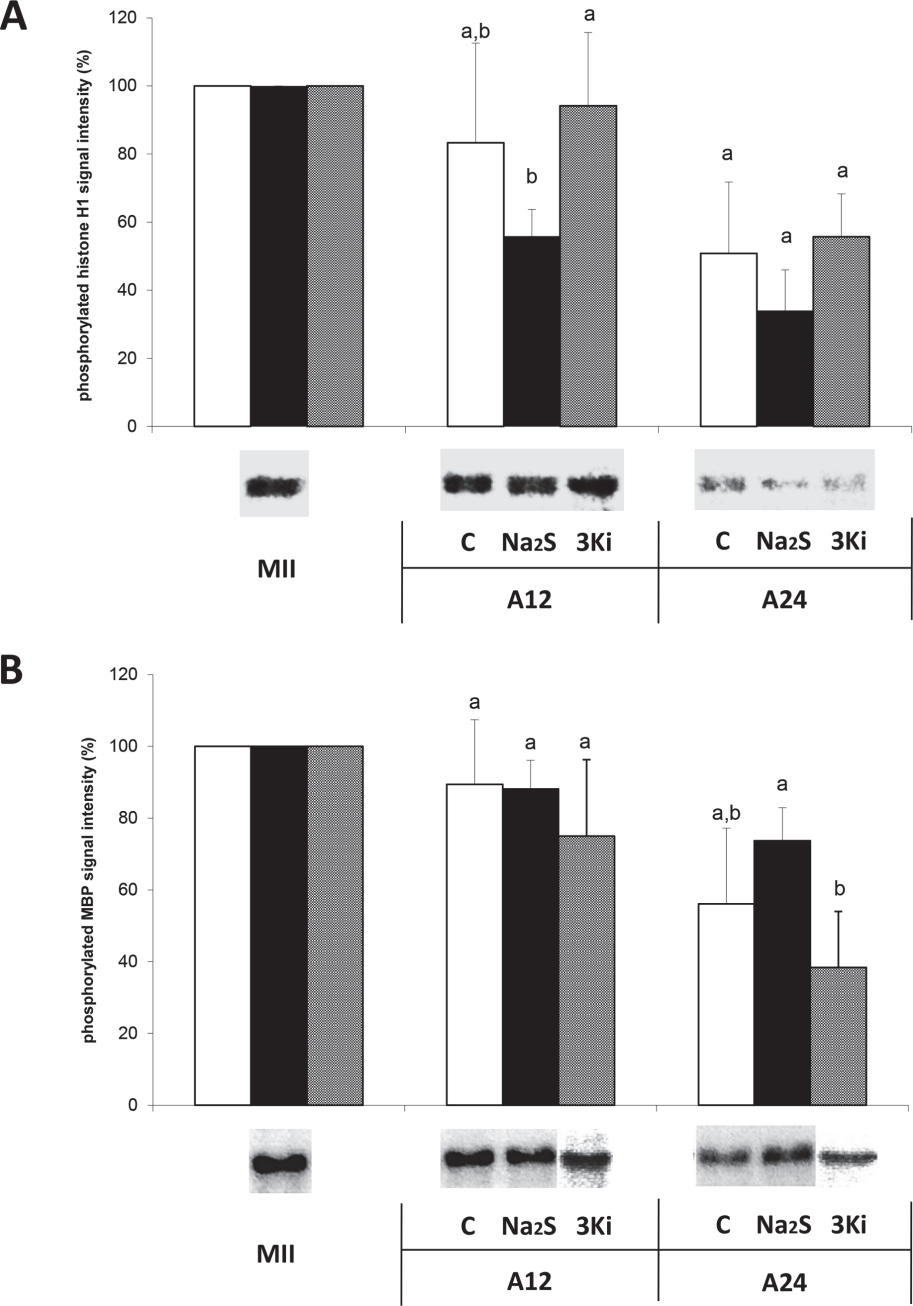
Effect of H_2_S donor on MPF and MAPK activity. 6A Histone H1 kinase assay was carried out to determine the activity of MPF by measurement of MPF capacity to phosphorylate its substrate (histone H1). **6B**: MBP kinase assay was carried out to determine the activity of MAPK by measurement of MAPK capacity to phosphorylate its substrate (MBP – Myelin basic protein). MPF and MAPK activities were determined in the MII oocytes (C – control, white column), the oocytes aged 12h and 24h in modified M199 medium, the oocytes aged 12h and 24h in modified M199 medium supplemented with a H_2_S donor (Na_2_S, black column), and the oocytes aged 12h and 24h in modified M199 medium supplemented with triple combination of inhibitors (3Ki, grey column). The results are presented as a ratio relative to the group of oocytes at metaphase II. *(GV – germinal vesicle stage; MII – oocytes at metaphase II; A12–12 hours of aging; A24–24 hours of aging; C – control, white column; Na_2_S—Na_2_S.9H_2_O, 300 μM, black column; 3Ki - 1mM oxamic acid + 1mM beta-kyano-L-alanine + 5mM alpha-ketoglutaric acid disodium salt dihydrate). ^a,b,^ Statistically significant differences in activity (MPF or MAPK) between individual treatments at the same time are indicated with different superscripts (P<0.05)*.

We monitored changes in the dynamics of key regulatory factors of meiotic maturation: MAPK and MPF. We observed a statistically significant decrease in the activity of MPF in oocytes that were cultured in the presence of H_2_S donor compared to those cultured in the presence of all three inhibitors of H_2_S-producing enzymes after 12 hours of aging (Fig. 6A). Conversely, MAPK activity decreased significantly after 24 hours of aging in oocytes that were cultured in the presence of inhibitors of H_2_S-producing enzymes compared to those cultured in the presence of the H_2_S donor (Fig. 6B).

### The H_2_S Donor Impairs the Efficiency of Induced Parthenogenetic Activation of Aged Porcine Oocytes

The next aim of this study was to evaluate the effect of exogenous H_2_S on the activating potential of aged oocytes. We wanted to investigate whether it was possible to parthenogenetically activate porcine oocytes that had been aged in the presence of elevated levels of H_2_S, with calcium ionophore and whether this treatment would have the same effect on their subsequent early embryonic development.

Following the parthenogenetic activation of oocytes that were not exposed to aging, 94.2 ± 5.2% of the oocytes were successfully activated (Table 1). Aging of oocytes for 24 hours led to a statistically significant decrease in the activation efficiency (to 79.2 ± 3.8%).

**Table 1:** Parthenogenetic activation of oocytes aged under the effect of the H_2_S donor.

**Groups**	**Na_2_S**	**Activated oocytes (%)**
**MII**	**0**	**94.2 ± 5.2^a^**
**24 hours**	**0**	**79.2 ± 3.8^b^**
**24 hours**	**150 μM**	**68.3 ± 3.8^b^**
**24 hours**	**300 μM**	**42.5 ± 5.0^c^**

At Oocytes were cultivated 48 hours to the metaphase II and then divided into 4 groups (see table). Control group (MII) was parthenogenetically activated immediately (without any exposure to prolonged cultivation). Other groups were exposed to prolonged cultivation (aging) for 24 hours in modified M199 medium supplemented with a H2S donor (Na_2_S.9H_2_O; 0μM, 150μM, and 300μM) and then parthenogenetically activated with calcium ionophore (25μM, 5 min) combined with 6-dimethyl aminopurine (2mM, 2 h). Subsequently, oocytes were cultured in NCSU 23 medium for the following 24 hours.

^a,b,c^ Statistically significant differences in the ratio of activated oocytes between individual treatments (in columns) are indicated with different superscripts (P<0.05).

The presence of an H_2_S donor did not increase the activation efficiency. Conversely, a statistically significant decrease in the portion of successfully activated oocytes was evident (to 42.5 ± 5.0) in the group of oocytes that was aged in medium supplemented with 300 μM of Na_2_S.9H_2_O.

### The H_2_S Donor Improves Embryonic Development Following Induced Parthenogenetic Activation of Aged Porcine Oocytes

In this experiment, we focused on the possibility of influencing the developmental competence of parthenogenetically activated aged oocytes by cultivating them in the presence of H_2_S donor during aging. The oocytes that were aged for 24 hours in medium supplemented with H_2_S donor (150 μM) showed significantly better early embryonic development in comparison to oocytes aged for 24 hours in medium without any supplementation. A significantly higher proportion of embryos reached the blastocyst stage. However, this ratio still did not reach the ratio of blastocysts observed in the control group, in which the oocytes were parthenogenetically activated in MII without exposure to the aging process. Higher concentrations of H_2_S donor (300 μM) did not have this positive effect (Table 2).

**Table 2:** Early embryonic development of parthenogenetically activated oocytes aged under the effect of the H_2_S donor.

**Groups**	**Na_2_S**	**Cleavage (%)**	**Morula (%)**	**Blastocyst (%)**
**MII**	**0**	**76.7 ± 3.8^a^**	**26.7 ± 2.9^a^**	**24.2± 1.4^a^**
**24 hours**	**0**	**41.7 ± 2.9^c^**	**8.3 ± 1.4^b^**	**1.7 ± 1.4^c^**
**24 hours**	**150 μM**	**54.2 ± 3.8^b^**	**20.8 ± 3.8^a^**	**15.8 ± 2.9^b^**
**24 hours**	**300 μM**	**26.7 ± 2.4^d^**	**13.3 ± 2.4^b^**	**3.3 ± 1.2^c^**

Oocytes were cultivated 48 hours to the metaphase II and then divided into 3 groups (see table). Control group (MII) was parthenogenetically activated immediately (without any exposure to prolonged cultivation). Other groups were exposed to prolonged cultivation (aging) for 24 hours in modified M199 medium supplemented with a H2S donor (Na_2_S.9H_2_O; 0μM, 150μM, and 300μM) and then parthenogenetically activated with calcium ionophore (25 μM, 5 min) combined with 6-dimethyl aminopurine (2 mM, 2 h). Subsequently, oocytes were cultured in NCSU 23 medium for 168 hours (7 days). The ratio of cleaved embryos was evaluated after the first 48 hours of culture.

^a,b,c,d^ Statistically significant differences in type of embryo stage between individual treatments (in columns) are indicated with different superscripts (P<0.05).

## Discussion

Although the gasotransmitter hydrogen sulfide has been recognized as a signal mediator, little is known about its involvement in oocyte physiology. In this study, we confirmed the role of hydrogen sulfide during the process of porcine oocyte aging in *in vitro* conditions. In a previous study, we demonstrated an effect of exogenous hydrogen sulfide (H_2_S) on the meiotic maturation of porcine oocytes, emphasizing that H_2_S could regulate oocyte meiosis as well as cumulus expansion [22]. The enzymes CBS, CSE and MPST are responsible for the endogenous production of H_2_S in somatic cells [32,33]. Although these enzymes have not yet been detected in mammalian oocytes, one can argue that in porcine oocytes, these enzymes are clearly active based on our results showing the formation of endogenous H_2_S and changes in its levels. This is the first evidence of endogenous H_2_S in porcine oocytes. In addition, we also demonstrated a reduction of endogenous H_2_S production as early as after 24 hours of the aging process of oocytes in *in vitro* conditions. Based on previous experiments, it is well known that, after this time point, morphological signs of oocyte aging are spontaneous parthenogenetic activation and, subsequently, the fragmentation or lysis of the oocytes [6].

Reduced H_2_S production may be either one of the biochemical markers accompanying aging or a hallmark of the aging process. The role of H_2_S in modulation of apoptosis on different somatic cells like lymphocytes, smooth muscle cells or fibroblast has been described [26]. Inhibition of endogenous H_2_S production leads to apoptosis, and sodium hydrosulfide as an exogenous donor of H_2_S can prevent apoptosis by improving mitochondrial dysfunction and suppressing the caspase-3 signaling pathway [34]. However, there is no evidence regarding the effect of H_2_S in mammalian oocytes.

We tested the hypothesis that H_2_S can play a role in aging by two means: first by sustaining and amplifying the decrease in H_2_S production; second, by artificially increasing H_2_S levels through the use of a hydrogen sulfide donor (Na_2_S).

Oxamic acid, beta-kyano-L-alanine and alpha-ketoglutaric acid disodium salt dihydrate were used as specific inhibitors of the activities of CBS, CSE and MPST [35, 36]. The use of these inhibitors led to an acceleration of porcine oocyte aging and significantly increased the proportion of fragmented oocytes during aging. The enzymes CBS and CSE are expected to be present in porcine oocyte based on the effects of their inhibition. These enzymes are major participants in the active formation of H_2_S in the reproductive tract [16], similarly to MPST, which has not yet been observed in the reproductive system of mammals.

It can be assumed that the increased proportion of apoptotic aged oocytes is largely caused by the decrease in endogenous H_2_S production because this effect can be reversed. Indeed, in the presence of H_2_S donor, a reversion of the negative effects of inhibitors was observed. Differing time courses of the aging process induced by CBS and CSE inhibition on the one hand and by MPST inhibition on the other, may reflect the specific roles of these enzymes in porcine oocytes or a distinct ability to compensate for the inhibition of one enzyme via other enzymes that were not affected by the specific inhibitors [37].

We also observed that addition of exogenous H_2_S via a hydrogen sulfide donor resulted in the suppression of oocyte fragmentation, which is a manifestation of apoptosis [38]. The selected H_2_S donor, Na_2_S, effectively suppressed apoptosis in aged porcine oocytes at concentrations comparable to physiological concentrations of H_2_S in tissues: the positive effect we observed at concentrations of Na_2_S.9H_2_O above 150 μM and, for example, in brain and in other tissues, occurred at concentrations of H_2_S that varied from 50 to 160 μM [14]. Based on these results, we hypothesize that the protective effect of an H_2_S donor against oocyte fragmentation might be due to the compensation for the decrease in endogenous H_2_S production that occurred spontaneously during the early stages of oocyte aging. Therefore, the influx of H_2_S from exogenous resources may contribute to the suppression of manifestations of some signs of aging in oocyte.

Two hallmarks of aging in mammalian oocytes are the decline in the activity of both MPF (M-phase promoting factor) and MAPK (mitogen-activated protein kinase). If these declines are prevented, then the ratio of fragmented oocytes decreases [39,40,41]. In our study, we did not observe significant changes in the dynamics of either MPF or MAPK activity under the influence of H_2_S donor at 12 and 24 hours of aging. The effect of an H_2_S donor apparently occurs via other regulatory mechanisms that remain to be determined. Another gasotransmitter, NO, appears to interfere with these pathways in an atypical manner. Jeseta et al. [42] described the parthenogenetic activation of *Xenopus laevis* oocytes in response to a nitric oxide donor, and this effect occurred in the absence of a dramatic effect on MPF activity. In the amphibian model, maintenance of MPF activity in metaphase-II-arrested oocytes has been shown to be required to trigger apoptosis [43]. Noticeably, a decrease in H_2_S levels beyond those detected during aging led to a drop in MAPK kinase activity after 24 hours but did not impact MPF. Based on our observations, it can be speculated that the MPF and MAPK pathways exhibit different sensitivities to changes in H_2_S changes that may be related to protein sulfhydration (e.g., MEK1, which is an activator of MAPK) [44]. Indeed, sulfhydration of proteins by H_2_S significantly alters the activity of these proteins [37]. For example, the activity of pro-apoptotic and anti-apoptotic factors can vary significantly under the influence of H_2_S [45]. Further studies are needed to characterize the members or regulators of these pathways that are modulated with respect to activities or location by sulfhydration.

Protection of oocytes against aging by increasing H_2_S levels might incur a cost. Indeed, in our culture conditions, the aged oocytes exhibited a reduced response to calcium ionophore treatment, which normally induces parthenogenetic activation. This response was even lower in groups of oocytes that were aged in the presence of an H_2_S donor. The causes of this decline are not yet known. Because parthenogenetic activation is a calcium-dependent process [46] and H_2_S has been reported to modulate calcium ion channel activity [47], we can hypothesize that the presence of an H_2_S donor impairs calcium signaling.

It is known that inadequate calcium signaling results in a reduced ratio of activated oocytes [48,49]. Surprisingly, however, a higher proportion of oocytes that had been aged in the presence of an H_2_S donor developed to the blastocyst stage following activation with an ionophore. However, hydrogen sulfide expression has a positive effect because the proportion of blastocysts and the developmental competence are quality markers of oocytes that have matured *in vitro*. The survival and development of the embryo depends heavily on mRNAs and proteins that accumulated during the growth and maturation of the oocyte. A majority of these products are used during the first embryonic divisions, after which transcriptional activation occurs in the embryonic genome. During oocyte aging, a range of processes occur that significantly disrupt developmental competency [50]. H_2_S appears to suppress at least some of these processes.

Although the intracellular mechanisms underlying the effect of H_2_S on biochemical processes in aged oocytes remain to be fully deciphered, our study clearly demonstrated a role for H_2_S in the protection of porcine oocytes against the aging process. Additional studies are needed to characterize the effects of H_2_S on survival, fertilization and early developmental processes in porcine oocytes.

## Supporting Information

S1 Table Effects of an elevated H_2_S level on porcine oocytes aging. Oocytes were cultivated to metaphase II and then exposed to prolonged cultivation in a modified M199 medium supplemented with a H_2_S donor for next 72 hours (Na_2_S.9H_2_O; 0μM, 75μM, 150μM, 300 μM, and 600 μM). ^*a,b,c*^ *Statistically signifficant differences in type of oocytes between individual concentrations of hydrogen sulfide donor (in columns) are indicated with different superscripts (P<0.05). The total number of oocytes in each experimental group was 120*.
(DOCX)

S2 Table Effects of an elevated H_2_S level and inhibition of H_2_S producing enzymes during oocyte aging - 24 hours. Oocytes were cultivated to metaphase II and then exposed to prolonged cultivation in a modified M199 medium for 24 hours in the presence of a H_2_S donor or H_2_S producing enzymes inhibitors. Na_2_S (Na_2_S.9H_2_O; 300 μM) was used as the H_2_S donor, oxamic acid (1mM, OA) was used as a CBS inhibitor, beta-kyano-L-alanine (1mM, KA) was used as a CSE inhibitor and alpha-ketoglutaric acid disodium salt dihydrate (5mM, KGA) was used as a MPST inhibitor. ^*a,b,c,d*^ *Statistically signifficant differences in type of oocytes between individual treatments (in columns) are indicated with different superscripts (P<0.05). The total number of oocytes in each experimental group was 120*.
(DOCX)

S3 Table Effects of an elevated H_2_S level and inhibition of H_2_S producing enzymes during oocyte aging - 48 hours. Oocytes were cultivated to metaphase II and then exposed to prolonged cultivation in a modified M199 medium for 48 hours in the presence of a H_2_S donor or H_2_S producing enzymes inhibitors. Na_2_S (Na_2_S.9H_2_O; 300 μM) was used as the H_2_S donor, oxamic acid (1mM, OA) was used as a CBS inhibitor, beta-kyano-L-alanine (1mM, KA) was used as a CSE inhibitor and alpha-ketoglutaric acid disodium salt dihydrate (5mM, KGA) was used as a MPST inhibitor. ^*a,b,c,d*^ *Statistically signifficant differences in type of oocytes between individual treatments (in columns) are indicated with different superscripts (P<0.05). The total number of oocytes in each experimental group was 120*.
(DOCX)

S4 Table Effects of an elevated H_2_S level and inhibition of H_2_S producing enzymes during oocyte aging - 72 hours. Oocytes were cultivated to metaphase II and then exposed to prolonged cultivation in a modified M199 medium for 72 hours in the presence of a H_2_S donor or H_2_S producing enzymes inhibitors. Na_2_S (Na_2_S.9H_2_O; 300 μM) was used as the H_2_S donor, oxamic acid (1mM, OA) was used as a CBS inhibitor, beta-kyano-L-alanine (1mM, KA) was used as a CSE inhibitor and alpha-ketoglutaric acid disodium salt dihydrate (5mM, KGA) was used as a MPST inhibitor. ^*a,b,c*^ *Statistically signifficant differences in type of oocytes between individual treatments (in columns) are indicated with different superscripts (P<0.05). The total number of oocytes in each experimental group was 120*.
(DOCX)

S5 Table Reversion of the effects of CBS, CSE and MPST inhibitors using a H_2_S donor. Oocytes were cultivated to metaphase II and then exposed to prolonged cultivation (24 hours) in a modified M199 medium supplemented with a H_2_S donor (Na_2_S.9H_2_O; 300 μM) and the following individual inhibitors: oxamic acid (1mM, OA), beta-kyano-L-alanine (1mM, KA), alpha-ketoglutaric acid disodium salt dihydrate (5mM, KGA), and its combination (see Table). *^a,b,c,d,e^* *Statistically signifficant differences in type of oocytes between individual treatments (in columns) are indicated with different superscripts (P<0.05). The total number of oocytes in each experimental group was 120*.
(DOCX)

S6 Table Effects of concurrent CBS, CSE and MPST inhibition and its reversion using a H_2_S donor - 24 hours of ageing. Oocytes were cultivated to metaphase II and then exposed to prolonged cultivation in a modified M199 medium supplemented with a H_2_S donor (Na_2_S.9H_2_O; 300 μM) and the inhibitors for 24 hours. Various combinations of oxamic acid (1mM, OA) which was used as a CBS inhibitor, beta-kyano-L-alanine (1mM, KA) which was used as a CSE inhibitor and alpha-ketoglutaric acid disodium salt dihydrate (5mM, KGA) which was used as a MPST inhibitor were used in this experiment. To reverse effects of inhibitors, a H_2_S donor (300 μM, Na_2_S.9H_2_O) was added to each experimental group. *^a,b,c^* *Statistically signifficant differences in type of oocytes between individual treatments (in columns) are indicated with different superscripts (P<0.05). The total number of oocytes in each experimental group was 120*.
(DOCX)

S7 Table Effects of concurrent CBS, CSE and MPST inhibition and its reversion using a H_2_S donor - 48 hours of ageing. Oocytes were cultivated to metaphase II and then exposed to prolonged cultivation in a modified M199 medium supplemented with a H_2_S donor (Na_2_S.9H_2_O; 300 μM) and the inhibitors for 48 hours. Various combinations of oxamic acid (1mM, OA) which was used as a CBS inhibitor, beta-kyano-L-alanine (1mM, KA) which was used as a CSE inhibitor and alpha-ketoglutaric acid disodium salt dihydrate (5mM, KGA) which was used as a MPST inhibitor were used in this experiment. To reverse effects of inhibitors, a H_2_S donor (300 μM, Na_2_S.9H_2_O) was added to each experimental group. *^a,b,c^* *Statistically signifficant differences in type of oocytes between individual treatments (in columns) are indicated with different superscripts (P<0.05). The total number of oocytes in each experimental group was 120*.
(DOCX)

S8 Table Effects of concurrent CBS, CSE and MPST inhibition and its reversion using a H_2_S donor - 72 hours of ageing. Oocytes were cultivated to metaphase II and then exposed to prolonged cultivation in a modified M199 medium supplemented with a H_2_S donor (Na_2_S.9H_2_O; 300 μM) and the inhibitors for 72 hours. Various combinations of oxamic acid (1mM, OA) which was used as a CBS inhibitor, beta-kyano-L-alanine (1mM, KA) which was used as a CSE inhibitor and alpha-ketoglutaric acid disodium salt dihydrate (5mM, KGA) which was used as a MPST inhibitor were used in this experiment. To reverse effects of inhibitors, a H_2_S donor (300 μM, Na_2_S.9H_2_O) was added to each experimental group. *^a,b,c^* *Statistically signifficant differences in type of oocytes between individual treatments (in columns) are indicated with different superscripts (P<0.05). The total number of oocytes in each experimental group was 120*.
(DOCX)
