# Supplementary material for: Hydrogen Sulfide Donor Protects Porcine Oocytes against Aging and Improves the Developmental Potential of Aged Porcine Oocytes
Source: PLoS One. 2015 Jan 23;10(1):e0116964. doi: 10.1371/journal.pone.0116964 (PMC4304783; doi:10.1371/journal.pone.0116964)
Supplement: S1 Table — Oocytes were cultivated to metaphase II and then exposed to prolonged cultivation in a modified M199 medium supplemented with a H2S donor for next 72 hours (Na2S.9H2O; 0μM, 75μM, 150μM, 300 μM, and 600 μM). a,b,c Statistically signifficant differences in type of oocytes between individual concentrations of hydrogen sulfide donor (in columns) are indicated with different superscripts (P<0.05). The total number of oocytes in each experimental group was 120. (DOCX) [file pone.0116964.s001.docx]

| **Na_2_S (μM)** | **Metaphase II (%)** | **Parthenotes (%)** | **Fragmented (%)** | **Lysed (%)** |
| --- | --- | --- | --- | --- |
| **0** | **21.7 ± 3.1^c^** | **46.7 ± 2.4^c^** | **26.6 ± 3.1^a^** | **5.0 ± 2.0^a^** |
| **75** | **19.2 ± 2.4^c^** | **50.8 ± 3.1^b^** | **25.8 ± 1.2^a^** | **4.2 ± 2.4^a^** |
| **150** | **36.7 ± 3.1^b^** | **63.3 ± 3.1^a^** | **0.0 ± 0.0^c^** | **0.0 ± 0.0^a^** |
| **300** | **55.8 ± 2.4^a^** | **44.2 ± 2.4^c^** | **0.0 ± 0.0^c^** | **0.0 ± 0.0^a^** |
| **600** | **31.7 ± 2.4^b^** | **58.3 ± 3.1^a^** | **9.2 ± 4.2^b^** | **0.8 ± 1.2^a^** |
