# Supplementary material for: Hydrogen Sulfide Donor Protects Porcine Oocytes against Aging and Improves the Developmental Potential of Aged Porcine Oocytes
Source: PLoS One. 2015 Jan 23;10(1):e0116964. doi: 10.1371/journal.pone.0116964 (PMC4304783; doi:10.1371/journal.pone.0116964)
Supplement: S2 Table — Oocytes were cultivated to metaphase II and then exposed to prolonged cultivation in a modified M199 medium for 24 hours in the presence of a H2S donor or H2S producing enzymes inhibitors. Na2S (Na2S.9H2O; 300 μM) was used as the H2S donor, oxamic acid (1mM, OA) was used as a CBS inhibitor, beta-kyano-L-alanine (1mM, KA) was used as a CSE inhibitor and alpha-ketoglutaric acid disodium salt dihydrate (5mM, KGA) was used as a MPST inhibitor. a,b,c,d Statistically signifficant differences in type of oocytes between individual treatments (in columns) are indicated with different superscripts (P<0.05). The total number of oocytes in each experimental group was 120. (DOCX) [file pone.0116964.s002.docx]

| **Treatment** | **Metaphase II (%)** | **Parthenotes (%)** | **Fragmented (%)** | **Lysed (%)** |
| --- | --- | --- | --- | --- |
| **0** | **99.2 ± 1.4^a^** | **0.9 ± 1.4^c^** | **0.0 ± 0.0^c^** | **0.0 ± 0.0^a^** |
| **Na_2_S** | **100.0 ± 0.0^a^** | **0.0 ± 0.0^c^** | **0.0 ± 0.0^c^** | **0.0 ± 0.0^a^** |
| **OA** | **71.7 ± 2.9^b^** | **6.6 ± 1.4^b^** | **21.7 ± 3.8^a^** | **0.0 ± 0.0^a^** |
| **KA** | **72.5 ± 2.5^b^** | **7.5 ± 2.5^b^** | **20.0 ± 2.5^a^** | **0.0 ± 0.0^a^** |
| **KGA** | **74.2 ± 2.9^b^** | **13.3 ± 2.9^a^** | **12.5 ± 0.0^b^** | **0.0 ± 0.0^a^** |
