# Supplementary material for: Hydrogen Sulfide Donor Protects Porcine Oocytes against Aging and Improves the Developmental Potential of Aged Porcine Oocytes
Source: PLoS One. 2015 Jan 23;10(1):e0116964. doi: 10.1371/journal.pone.0116964 (PMC4304783; doi:10.1371/journal.pone.0116964)
Supplement: S4 Table — Oocytes were cultivated to metaphase II and then exposed to prolonged cultivation in a modified M199 medium for 72 hours in the presence of a H2S donor or H2S producing enzymes inhibitors. Na2S (Na2S.9H2O; 300 μM) was used as the H2S donor, oxamic acid (1mM, OA) was used as a CBS inhibitor, beta-kyano-L-alanine (1mM, KA) was used as a CSE inhibitor and alpha-ketoglutaric acid disodium salt dihydrate (5mM, KGA) was used as a MPST inhibitor. a,b,c Statistically signifficant differences in type of oocytes between individual treatments (in columns) are indicated with different superscripts (P<0.05). The total number of oocytes in each experimental group was 120. (DOCX) [file pone.0116964.s004.docx]

| **Treatment** | **Metaphase II (%)** | | | **Parthenotes (%)** | **Fragmented (%)** | | **Lysed (%)** | |
| --- | --- | --- | --- | --- | --- | --- | --- | --- |
| **0** | | **23.3 ± 1.4^b^** | **47.5 ± 2.5^a^** | | **25.9 ± 2.9^b^** | **3.3 ± 1.4^a^** | |  |
| **Na_2_S** | | **55.0 ± 5.0^a^** | **45.0 ± 5.0^a^** | | **0.0 ± 0.0^c^** | **0.0 ± 0.0^a^** | |  |
| **OA** | | **20.9 ± 3.8^b^** | **41.7 ± 7.6^a^** | | **33.3 ± 2.9^b^** | **4.1 ± 1.4^a^** | |  |
| **KA** | | **21.7 ± 1.4^b^** | **46.7 ± 2.9^a^** | | **28.3 ± 1.4^b^** | **3.3 ± 1.4^a^** | |  |
| **KGA** | | **25.0 ± 4.9^b^** | **22.0 ± 3.5^b^** | | **51.3 ± 4.3^a^** | **1.7 ± 1.4^a^** | |  |
