# Supplementary material for: Hydrogen Sulfide Donor Protects Porcine Oocytes against Aging and Improves the Developmental Potential of Aged Porcine Oocytes
Source: PLoS One. 2015 Jan 23;10(1):e0116964. doi: 10.1371/journal.pone.0116964 (PMC4304783; doi:10.1371/journal.pone.0116964)
Supplement: S5 Table — Oocytes were cultivated to metaphase II and then exposed to prolonged cultivation (24 hours) in a modified M199 medium supplemented with a H2S donor (Na2S.9H2O; 300 μM) and the following individual inhibitors: oxamic acid (1mM, OA), beta-kyano-L-alanine (1mM, KA), alpha-ketoglutaric acid disodium salt dihydrate (5mM, KGA), and its combination (see Table). a,b,c,d,e Statistically signifficant differences in type of oocytes between individual treatments (in columns) are indicated with different superscripts (P<0.05). The total number of oocytes in each experimental group was 120. (DOCX) [file pone.0116964.s005.docx]

| **Inhibitor** | **Donor** | | **Metaphase II (%)** | | **Parthenotes (%)** | | **Fragmented (%)** | | **Lysed (%)** |
| --- | --- | --- | --- | --- | --- | --- | --- | --- | --- |
| ***-*** | | ***-*** | | **100.0 ± 0.0^a^** | | **0.0 ± 0.0^e^** | | **0.0 ± 0.0^c^** | **0.0 ± 0.0^a^** |
| ***-*** | | **Na_2_S** | | **100.0 ± 0.0^a^** | | **0.0 ± 0.0^e^** | | **0.0 ± 0.0^c^** | **0.0 ± 0.0^a^** |
| **OA** | | **-** | | **70.0 ± 2.5^b^** | | **8.3 ± 1.4^c^** | | **21.7 ± 1.4^a^** | **0.0 ± 0.0^a^** |
| **OA** | | **Na_2_S** | | **94.2 ± 1.4^a^** | | **4.2 ± 1.4^d,e^** | | **0.8 ± 1.4 ^c^** | **0.8 ± 1.4^a^** |
| **KA** | | **-** | | **71.7 ± 2.9^b^** | | **5.8 ± 1.4^c,d^** | | **22.5 ± 2.5^a^** | **0.0 ± 0.0^a^** |
| **KA** | | **Na_2_S** | | **97.5 ± 2.5^a^** | | **1.7 ± 1.4^e^** | | **0.8 ± 1.4^c^** | **0.0 ± 0.0^a^** |
| **KGA** | | **-** | | **76.7 ± 2.9^b^** | | **11.6 ± 2.9^b^** | | **11.7 ± 5.8^b^** | **0.0 ± 0.0^a^** |
| **KGA** | | **Na_2_S** | | **78.3 ± 1.4^b^** | | **20.0 ± 0.0^a^** | | **1.7 ± 1.4^c^** | **0.0 ± 0.0^a^** |
