# Supplementary material for: Hydrogen Sulfide Donor Protects Porcine Oocytes against Aging and Improves the Developmental Potential of Aged Porcine Oocytes
Source: PLoS One. 2015 Jan 23;10(1):e0116964. doi: 10.1371/journal.pone.0116964 (PMC4304783; doi:10.1371/journal.pone.0116964)
Supplement: S8 Table — Oocytes were cultivated to metaphase II and then exposed to prolonged cultivation in a modified M199 medium supplemented with a H2S donor (Na2S.9H2O; 300 μM) and the inhibitors for 72 hours. Various combinations of oxamic acid (1mM, OA) which was used as a CBS inhibitor, beta-kyano-L-alanine (1mM, KA) which was used as a CSE inhibitor and alpha-ketoglutaric acid disodium salt dihydrate (5mM, KGA) which was used as a MPST inhibitor were used in this experiment. To reverse effects of inhibitors, a H2S donor (300 μM, Na2S.9H2O) was added to each experimental group. a,b,c Statistically signifficant differences in type of oocytes between individual treatments (in columns) are indicated with different superscripts (P<0.05). The total number of oocytes in each experimental group was 120. (DOCX) [file pone.0116964.s008.docx]

| **Treatment** | **Metaphase II (%)** | | | **Parthenotes (%)** | **Fragmented (%)** | | **Lysed (%)** | |
| --- | --- | --- | --- | --- | --- | --- | --- | --- |
| **0** | | **25.8 ± 2.9^c^** | **44.2 ± 3.8^b^** | | **26.7 ± 3.8^c^** | **3.3 ± 1.4^a^** | |  |
| **Na_2_S** | | **53.3 ± 2.9^a^** | **46.7 ± 2.9^b^** | | **0.0 ± 0.0^e^** | **0.0 ± 0.0^a^** | |  |
| **OA + KA** | | **41.7 ± 1.4^b^** | **32.5 ± 2.5^c^** | | **25.8 ± 3.8^c^** | **0.0 ± 0.0^a^** | |  |
| **OA + KA + Na_2_S** | | **43.0 ± 4.0^a,b^** | **56.2 ± 3.8^a^** | | **0.0 ± 0.0^e^** | **0.8 ± 1.4^a^** | |  |
| **OA + KGA** | | **38.3 ± 1.4^b^** | **33.3 ± 1.4^c^** | | **24.2 ± 3.8^c^** | **4.2 ± 2.9^a^** | |  |
| **OA + KGA+ Na_2_S** | | **51.1 ± 5.1^a^** | **32.2 ± 5.1^c^** | | **10.0 ± 0.0^d^** | **6.7 ± 0.0^a^** | |  |
| **KA + KGA** | | **20.0 ± 2.5^d^** | **30.8 ± 1.4^c^** | | **49.2 ± 1.4^b^** | **0.0 ± 0.0^a^** | |  |
| **KA + KGA+ Na_2_S** | | **45.8 ± 3.8^a,b^** | **54.2 ± 3.8^a^** | | **0.0 ± 0.0^e^** | **0.0 ± 0.0^a^** | |  |
| **OA + KA + KGA** | | **10.0 ± 5.0^e^** | **24.2 ± 2.9^d^** | | **65.8 ± 2.9^a^** | **0.0 ± 0.0^a^** | |  |
| **OA + KA + KGA+ Na_2_S** | | **22.9 ± 3.1^c,d^** | **53.3 ± 3.8^a^** | | **20.5 ± 4.0^c^** | **3.3 ± 1.3^a^** | |  |
